# The reference genome of a Sierra Nevada endemic, the cut-leaved monkeyflower, *Mimulus laciniatus* (syn. *Erythranthe lacinata*)

**DOI:** 10.1093/jhered/esaf059

**Published:** 2025-08-28

**Authors:** Jesús Martínez-Gómez, Merly Escalona, Jack M Colicchio, Lauren N Hamm, Mohan P A Marimuthu, Oanh Nguyen, Noravit Chumchim, William Seligmann, Rachel S Meyer, Jason P Sexton, Benjamin K Blackman

**Affiliations:** Department of Plant & Microbial Biology, University of California Berkeley, Berkeley, CA, United States; Department of Integrative Biology, University of California Berkeley, Berkeley, CA, United States; Department of Biomolecular Engineering, University of California Santa Cruz, Santa Cruz, CA, United States; Department of Plant & Microbial Biology, University of California Berkeley, Berkeley, CA, United States; Department of Plant & Microbial Biology, University of California Berkeley, Berkeley, CA, United States; DNA Technologies and Expression Analysis Core Laboratory, Genome Center, University of California, Davis, CA, United States; DNA Technologies and Expression Analysis Core Laboratory, Genome Center, University of California, Davis, CA, United States; DNA Technologies and Expression Analysis Core Laboratory, Genome Center, University of California, Davis, CA, United States; Department of Ecology & Evolutionary Biology, University of California Santa Cruz, Santa Cruz, CA, United States; Department of Ecology & Evolutionary Biology, University of California Santa Cruz, Santa Cruz, CA, United States; Department of Life and Environmental Sciences, University of California Merced, Merced, CA, United States; Department of Plant & Microbial Biology, University of California Berkeley, Berkeley, CA, United States; Department of Integrative Biology, University of California Berkeley, Berkeley, CA, United States

**Keywords:** California Conservation Genomics Project, California endemic plants, CCGP, conservation, monkeyflower, speciation

## Abstract

*Mimulus laciniatus* (syn. *Erythranthe lacinata*) is an annual plant endemic to the Sierra Nevada region of California. *M. laciniatus* is notable for its specialized ecological niche, thriving in granite outcrops of alpine environments characterized by shallow soils that dry out rapidly as the snowpack is exhausted during season-ending droughts. Due to its narrow habitat range and sensitivity to environmental change, this species serves as an important model for studying adaptation and survival in marginal habitats. As part of the California Conservation Genomics Project, here we report the sequencing and assembly of a high-quality nuclear genome and chloroplast genome of *M. laciniatus*. The primary assembly is 309.97 Mb and consists of 104 scaffolds with a scaffold N50 of 20.99 Mb, a largest contig size of 24.29 Mb, and a contig N50 of 11.09 Mb, The alternate haplotype assembly consists of 194 scaffolds spanning 213.84 Mb. BUSCO completeness of the primary assembly is 98.6%. This high quality genome adds a valuable resource to the expanding collection of sequenced genomes of the monkeyflowers (*Mimulus* sensu lato), which have become a model clade for studying ecological adaptation, speciation, and evolutionary genetics.

## Introduction

A foundational goal in evolutionary biology, ecology, and conservation biology is to understand the processes that cause lineages to speciate, persist, and go extinct. The neoendemic plants of the California floristic province (CA-FP) are an excellent system to study the processes that create, maintain, and diminish biodiversity. The CA-FP is the only member of the 36 globally recognized biodiversity hotspots (Mittermeier et al. 2011) located in the continental United States and Canada (reviewed in Baldwin 2014). Endemic species—i.e. species with a distribution restricted to a particular geographic range—are an important contributor to the biodiversity of the CA-FP. Of the 6143 minimally ranked vascular plant taxa (i.e. species, subspecies, varieties) native to the CA-FP, 42% are endemic to the CA-FP (Baldwin and Goldman 2012, Burge et al. 2016). Furthermore, more than half of the endemics (63.6%) have an annual life history (Raven and Axelrod 1974). Endemic plants of California have been used as models to understand speciation, plasticity, and local adaptation (Hodges et al. 2004, Hufford and Mazer 2012, Pettengill and Moeller 2012, Gould et al. 2014, Sobel 2014, Hernández and Specht 2023), and understanding the mechanisms that shape diversity is necessary to develop conservation plans for endemics.

The yellow monkeyflower species complex *Mimulus* sect. *Simiolus* (syn. *Erythranthe* sect. *Simiola*) and the larger clade of monkeyflowers as a whole (reviewed in Yuan 2019), have long served as model lineages to study the evolutionary and ecological processes that drive adaptation and speciation (Vickery 1966, Wu et al. 2008, Twyford et al. 2015). The yellow monkeyflower clade has drawn interest due to its striking variation, including the colonization of diverse stressful edaphic environments (MacNair 1983, MacNair 1993, Lekberg et al. 2012, Sianta and Kay 2019), mating system evolution (Fenster and Ritland 1994, Martin and Willis 2007, Fishman et al. 2015), and transitions between annual and perennial life history strategies (Lowry et al. 2008, Coughlan et al. 2021). Members of this group are particularly well suited for the study of narrowly distributed taxa, especially those endemic to the CA-FP. About half of the ca. 50 minimally ranked monkeyflower taxa in sect. *Simiolus* are narrowly distributed (Nesom 2014), and 9 of these are endemic to CA-FP (Table S1).

**Fig. 1:**
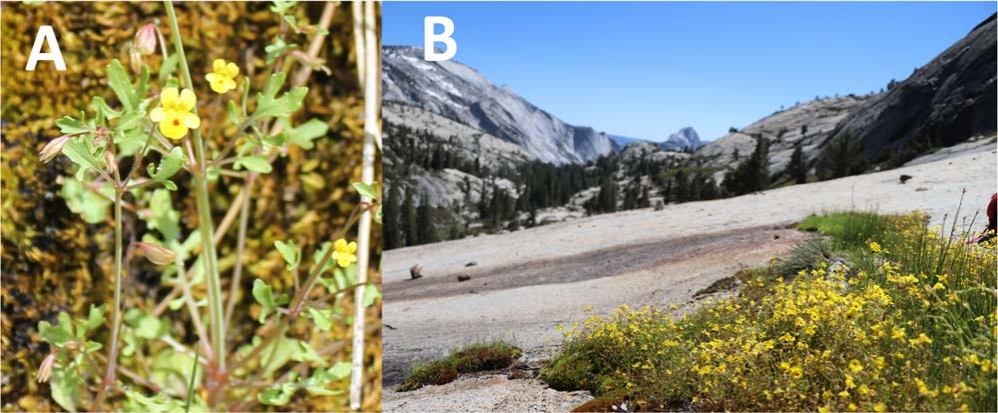
*Mimulus laciniatus* and its growth habit at Olmstead point. A) *M. laciniatus* inflorescence, displaying dissected leaves (photo credit: Benjamin Blackman). B) Source population of sequenced individual OPN: Olmstead point in Yosemite National Park, Mariposa County CA (photo credit: Diana Tataru).

*Mimulus laciniatus* A. Gray (syn. *Erythranthe laciniata* (A. Gray) G.L. Nesom) has garnered special attention in the study of narrowly distributed taxa (Fig. 1A; Note: *Erythranthe laciniata* is the appellation in the most recent taxonomic revision of this group (Barker et al. 2012)*.* We continue to apply the older taxonomy for consistency with prior studies, predominance of use in the research community (Lowry et al. 2019), and public genomic resources including additional monkeyflower reference genome assemblies (Goodstein et al. 2012)). *Mimulus laciniatus* is restricted to the western slope of the Sierra Nevada and their western foothills. Across its range, *M. laciniatus* can be found in a wide range of climates and habitats (i.e. foothill woodland, montane mixed-conifer, subalpine, and alpine communities) and spans a broad elevational range (~900 to 3,270 m), but populations are generally restricted to seeps on granite outcrops, often co-occurring with mosses and spikemosses (Sexton and Dickman 2016, Fraga 2018; Fig. 1B). *Mimulus laciniatus* is largely self-fertilizing, reflected by its low observed heterozygosity (h_o_ = 0.07; Ferris et al. 2014) and high inbreeding coefficient (F_IS_ = 0.93; Sexton et al. 2016). The taxon is distinguished by its dissected leaves that differentiate it from nearly all other closely related members of the species complex except *Mimulus filicifolius* (syn. *Erythranthe filicifolia*; Sexton et al. 2013) and rare populations of *Mimulus guttatus* (syn. *E. guttata* and *E. microphylla*; Ferris et al. 2015). However, in the lab, *M. laciniatus* can readily be crossed with other members of the complex, and the presence of natural hybrid zones indicates low or lack of intrinsic reproductive barriers (Vickery 1978, Tataru et al. 2025). Given the degree of morphological and ecological differentiation with respect to the widely distributed outcrosser *M. guttatus*, this taxon has been used to study traits related to adaptive divergence and reproductive isolation, specifically the genetic basis of flowering time and its plasticity to environmental cues (Friedman and Willis 2013, Ferris 2016, Leinonen et al. 2020), leaf shape (Ferris et al. 2015, Ferris et al. 2017), and floral morphology (Ferris et al. 2017). Many of these traits are hypothesized or shown to be adaptive and contribute to local adaptation of the species (DeMarche et al. 2013, Ferris and Willis 2018, Tataru et al. 2023).

In addition to this work on the ecology and genetics of speciation and adaptation, *M. laciniatus* has provided insight into the factors that limit species distributions (i.e. range limits) and on conservation strategies for narrow endemics of the CA-FP. Research has demonstrated that populations at the warmer edges of the species range (i.e. foothill habitats) benefit significantly from gene flow originating from populations occupying similar warm-edge climates (Sexton et al. 2011). Conversely, gene flow from centrally located populations (i.e. montane habitats) has been shown to have a detrimental effect, indicating the importance of the gene flow source. Moreover, patterns of genetic isolation by climate environment, rather than isolation by distance, have been found across the species range (Sexton et al. 2016). This finding underscores the importance of carefully evaluating spatial genetic relationships when developing conservation strategies, such as assisted gene flow programs to promote genetic or evolutionary rescue. Additionally, studies investigating adaptive responses to climate change have revealed substantial genetic potential for drought tolerance within this group (Dickman et al. 2019). However this resilience is relative. Compared to the widely distributed *M. guttatus*, *M. laciniatus* tolerates a narrower range of temperatures—a pattern consistent with other monkeyflower species pairs where distribution breath correlates with thermal tolerance (Sheth et al. 2014; Sheth and Angert 2014). The contrast between widely distributed species and narrowly distributed species highlights the distinct dynamics and unique vulnerabilities of narrowly distributed species, reinforcing the need for targeted study and conservation strategies.

Genomics has been instrumental in advancing many of these studies, providing critical insights and enabling novel approaches to answering fundamental questions in evolutionary biology, ecology, and conservation. For instance, DNA sequencing supports efforts to map the genetic basis of adaptive trait variation, infer evolutionary relationships, and provide necessary context and tools for conservation strategies. Prior genomic studies have largely been performed by aligning *M. laciniatus* sequences to a *M. guttatus* genome assembly as a reference, but reference genome bias, gene presence-absence variation, and structural variation create caveats or limitations that can impact results (Günther and Nettelblad 2019). Here we report a haplotype resolved genome of *M. laciniatus.* Produced as a part of the California Genomics Conservation Project (Fiedler et al*.* 2022, Shaffer et al. 2022) and used in concert with additional resources for more broadly distributed taxa in *Mimulus* sect. *Simiolus* (Lovell et al. 2025; Kollar et al. 2025), this genome will empower new, more rigorous research into how annual endemics of the CA-FP evolve and how to develop effective conservation strategies for these taxa.

## Methods

### Biological material

Dried fruits were collected from *M. laciniatus* located at site OPN (Olmstead Point; 37.81073°N, 119.48518°W; Elev. 2,530 m) in Yosemite National Park, Mariposa County, California (Leinonen et al. 2020). Seeds from this individual were grown in the lab and selfed for multiple generations; tissue was collected from a single descendant of this line. Multiple floral buds were collected in a 50 mL conical tube and frozen.

### Nucleic acid library preparation

#### PacBio HiFi

We extracted high molecular weight (HMW) genomic DNA (gDNA) from 130 mg of buds using the cetyltrimethylammonium bromide (CTAB) method as described in Inglis et al. (2018), with the following modifications: 1) we used sodium metabisulfite (1% w/v) instead of 2-mercaptoethanol (1% v/v) in the sorbitol wash buffer and CTAB solution; 2) we repeated the tissue homogenate wash steps until the supernatant turned clear; 3) we performed the CTAB lysis step at 45 °C; and 4) the chloroform extraction step twice using ice-cold chloroform. The DNA purity was estimated by using the NanoDrop ND-1000 spectrophotometer (Thermo Fisher Scientific, Waltham, MA). The DNA yield (1.3 μg) was quantified using a Quantus Fluorometer (QuantiFluor ONE dsDNA Dye assay; Promega, Madison, WI), and the size distribution of the DNA was estimated using the Femto Pulse system (Genomic DNA 165 kb kit, Agilent, Santa Clara, CA), where 70% of the DNA fragments were found to be 30 kilobases (kb) or longer.

The HiFi SMRTbell library was constructed using the SMRTbell prep kit 3.0 (Pacific Biosciences—PacBio, Menlo Park, CA; Cat. #102-182-700) according to the manufacturer’s instructions. HMW gDNA was sheared to a target DNA size distribution between 15 and 18 kb using Diagenode’s Megaruptor 3 system (Diagenode, Belgium; cat. B06010003). The sheared gDNA was concentrated using 1× of SMRTbell cleanup beads provided in the SMRTbell prep kit 3.0 for the repair and a-tailing incubation at 37 °C for 30 min and 65 °C for 5 min, followed by ligation of overhang adapters at 20 °C for 30 min, clean-up using 1X SMRTbell cleanup beads, and nuclease treatment at 37 °C for 15 min. The SMRTbell library was size selected using 3.1X of 35% v/v diluted AMPure PB beads (PacBio, Cat. #100-265-900) to progressively remove SMRTbell templates < 5 kb. The 15 to 18 kb average HiFi SMRTbell library was sequenced at UC Davis DNA Technologies Core (Davis, CA) using one 8 M SMRT cell (PacBio, Cat #101-389-001), Sequel II sequencing chemistry 2.0, and 30-h movies each on a PacBio Sequel IIe sequencer.

### Omni-C

The Omni-C library was prepared using the Dovetail™ Omni-C™ Kit (Dovetail Genomics, Scotts Valley, CA) according to the manufacturer’s protocol with slight modifications. First, tissue from floral buds and meristems was thoroughly ground with a mortar and pestle while cooled with liquid nitrogen. Nuclear isolation was then performed using published methods (Workman et al. 2018). Subsequently, chromatin was fixed in place in the nucleus and digested under various conditions of DNase I until a suitable fragment length distribution of DNA molecules was obtained. Chromatin ends were repaired and ligated to a biotinylated bridge adapter followed by proximity ligation of adapter containing ends. After proximity ligation, crosslinks were reversed and the DNA was purified from proteins. Purified DNA was treated to remove biotin that was not internal to ligated fragments. A next-generation sequencing library was generated using an NEB Ultra II DNA Library Prep kit (New England Biolabs, Ipswich, MA) with an Illumina compatible y-adaptor. Biotin-containing fragments were then captured using streptavidin beads. The post-capture product was split into two replicates prior to PCR enrichment to preserve library complexity with each replicate receiving unique dual indices. The library was sequenced at Vincent J. Coates Genomics Sequencing Lab (Berkeley, CA) on an Illumina NovaSeq 6000 platform (Illumina, CA) to generate approximately 100 million 2 × 150 bp read pairs per gigabase (Gb) of genome size.

### Nuclear genome assembly

We assembled the genome of a *M. laciniatus* individual following the CCGP assembly pipeline Version 5.0, as outlined in Table 1 listing the tools and non-default parameters used in the assembly process. We removed the remnants of adapter sequences from the PacBio HiFi dataset using HiFiAdapterFilt (Sim et al. 2022) and generated an initial diploid phased assembly using HiFiasm (Cheng et al. 2022) in HiC mode, with the filtered PacBio HiFi reads and the Omni-C short-reads, a process that generates two assemblies, one per haplotype. We aligned the Omni-C data to each assembly following the Arima Genomics Mapping Pipeline (https://github.com/ArimaGenomics/mapping_pipeline) and then scaffolded the assemblies with SALSA (Ghurye et al. 2017, Ghurye et al. 2019).

**Table 1:** Assembly pipeline and software used.

**Assembly step**	**Software and any non-default options**	**Version**	**Reference**	
**Initial assembly**				
Filtering PacBio HiFi adapters	HiFiAdapterFilt	Commit 64d1c7b	Sim et al. 2022	
**K-mer counting**	Meryl (k = 21)	1	https://github.com/marbl/meryl	
**Estimation of genome size and heterozygosity**	GenomeScope (−l.50)	2	Ranallo-Benavidez et al. 2020	
***De novo assembly (contiging)***	HiFiasm (Hi-C Mode, -l0, –primary, output hic.hap1.p_ctg, hic.hap2.p_ctg)	0.19.5-r592	Cheng et al. 2022	
**Scaffolding**				
**Omni-C data alignment**	Arima Genomics Mapping Pipeline	Commit 2e74ea4	https://github.com/ArimaGenomics/mapping_pipeline	
**Arima Genomics Mapping Pipeline (AGMP)**	BWA-MEM	0.7.17-r1188	Li 2013	
	samtools	1.11	Danecek et al. 2021	
	filter_five_end.pl (AGMP)	Commit 2e74ea4	https://github.com/ArimaGenomics/mapping_pipeline	
	two_read_bam_combiner.pl (AGMP)	Commit 2e74ea4	https://github.com/ArimaGenomics/mapping_pipeline	
	picard	2.27.5	https://broadinstitute.github.io/picard/	
**Omni-C Scaffolding**	SALSA (-DNASE, -i 20, -p yes)	2	Ghurye et al. 2017, Ghurye et al. 2019	
**Omni-C Contact map generation**				
**Short-read alignment**	BWA-MEM (-5SP)	0.7.17-r1188	Li 2013	
**SAM/BAM processing**	samtools	1.11	Danecek et al. 2021	
**SAM/BAM filtering**	pairtools	0.3.0	Open2C et al. 2024	
**Pairs indexing**	pairix	0.3.7	Lee et al. 2022	
**Matrix generation**	cooler	0.8.10	Abdennur and Mirny 2020	
**Matrix balancing**	hicExplorer (hicCorrectmatrix correct --filterThreshold −2 4)	3.6	Ramírez et al. 2018	
**Contact map visualization**	HiGlass	2.1.11	Kerpedjiev et al. 2018	
	PretextMap	0.1.4	https://github.com/wtsi-hpag/PretextView	
	PretextView	0.1.5	https://github.com/wtsi-hpag/PretextMap	
	PretextSnapshot	0.0.3	https://github.com/wtsi-hpag/PretextSnapshot	
**Manual curation tools**	Rapid curation pipeline (Wellcome Trust Sanger Institute, Genome Reference Informatics Team)	Commit 7acf220c	https://gitlab.com/wtsi-grit/rapid-curation	
**Genome quality assessment**				
**Basic assembly metrics**	QUAST (--est-ref-size)	5.0.2	Gurevich et al. 2013	
**Assembly completeness**	BUSCO (-m geno, -l embryophyta)	5.0.0	Manni et al. 2021	
	Merqury	2020 January 29	Rhie et al. 2020	
**Contamination screening**				
**Local alignment tool**	BLAST+ (-db nt, -outfmt ‘6 qseqid staxids bitscore std’, -max_target_seqs 1, -max_hsps 1, -evalue 1e-25)	2.15	Camacho et al. 2009	
**General contamination screening**	BlobToolKit (HiFi coverage, BUSCO = embryophyta, NCBI Taxa ID = 4156)	2.3.3	Challis et al. 2020	
**Chloroplast genome assembly**				
***de novo* assembler of organelle genomes**	Oatk	1.0 (Commit 6591c42)	https://github.com/c-zhou/oatk	
**Organeller genome annotation**	GeSeq	https://chlorobox.mpimp-golm.mpg.de/geseq.html	Tillich et al. 2017	

The assemblies for both haplotypes were manually curated by iteratively generating and analyzing their corresponding Omni-C contact maps. Briefly, to generate the contact maps we aligned the Omni-C data with BWA-MEM (Li 2013), identified ligation junctions, and generated Omni-C pairs (Lee et al. 2022) using pairtools (Open2C et al. 2024). Then, we generated multi-resolution Omni-C matrices with Cooler (Abdennur and Mirny 2020) and balanced them with hicExplorer (Ramírez et al. 2018). We used HiGlass (Kerpedjiev et al. 2018) and the PretextSuite (https://github.com/wtsi-hpag/PretextView; https://github.com/wtsi-hpag/PretextMap; https://github.com/wtsi-hpag/PretextSnapshot) to visualize the contact maps. We identified misassemblies and misjoins in these contact maps, and modified the assemblies using the Rapid Curation pipeline from the Wellcome Trust Sanger Institute, Genome Reference Informatics Team (https://gitlab.com/wtsi-grit/rapid-curation). Some of the remaining gaps (joins generated during scaffolding and/or curation) were closed using the PacBio HiFi reads and YAGCloser (https://github.com/merlyescalona/yagcloser). We checked for contamination using the BlobToolKit Framework (Challis et al. 2020).

### Genome quality assessment

We generated k-mer counts from the Omni-C short-read and PacBio HiFi long-read datasets. For the Omni-C short read data, we first removed adapters using cutadapt (Martin 2011), following the indications from Dovetail Genomics (https://omni-c.readthedocs.io/en/latest/assembly.html). For the long-read data, we used the adapter-trimmed dataset. Then, we generated the k-mer counts for each dataset using meryl (https://github.com/marbl/meryl). The k-mer counts were then used in GenomeScope2.0 (Ranallo-Benavidez et al. 2020) to estimate genome features including genome size, heterozygosity, and repeat content. To obtain general contiguity metrics, we ran QUAST (Gurevich et al. 2013). To evaluate genome quality and functional completeness, we used BUSCO (Manni et al. 2021) with the Embryophyta ortholog database (embryophyta_odb10) which contains 1,614 genes. Assessment of base level accuracy (QV) and k-mer completeness was performed using the previously generated meryl database and merqury (Rhie et al. 2020). We further estimated genome assembly accuracy via BUSCO gene set frameshift analysis using the pipeline described in Korlach et al. (2017). Measurements of the size of the phased blocks is based on the size of the contigs generated by HiFiasm on HiC mode. We follow the quality metric nomenclature established by Rhie et al. (2021), with the genome quality code x.y.P.Q.C, where, x = log10[contig NG50]; y = log10[scaffold NG50]; P = log10 [phased block NG50]; Q = Phred base accuracy QV (quality value); C = % genome represented by the first “n” scaffolds, following a karyotype of 2n = 28 for this species (Vickery 1995). Quality metrics for the notation were calculated on the primary haplotype assembly.

### Chloroplast genome assembly

The chloroplast sequence for *M. laciniatus* was generated with Oatk (https://github.com/c-zhou/oatk). We used GeSeq (Tillich et al. 2017) to generate a draft genome annotation. After completion of the nuclear genome, we searched for matches of the resulting chloroplast assembly sequence in the nuclear genome assembly using BLAST+ (Camacho et al. 2009) and filtered out contigs and scaffolds from the nuclear genome with a percentage of sequence identity > 99% and size smaller than the chloroplast assembly sequence.

## Results

### Sequencing results

**Fig. 2:**
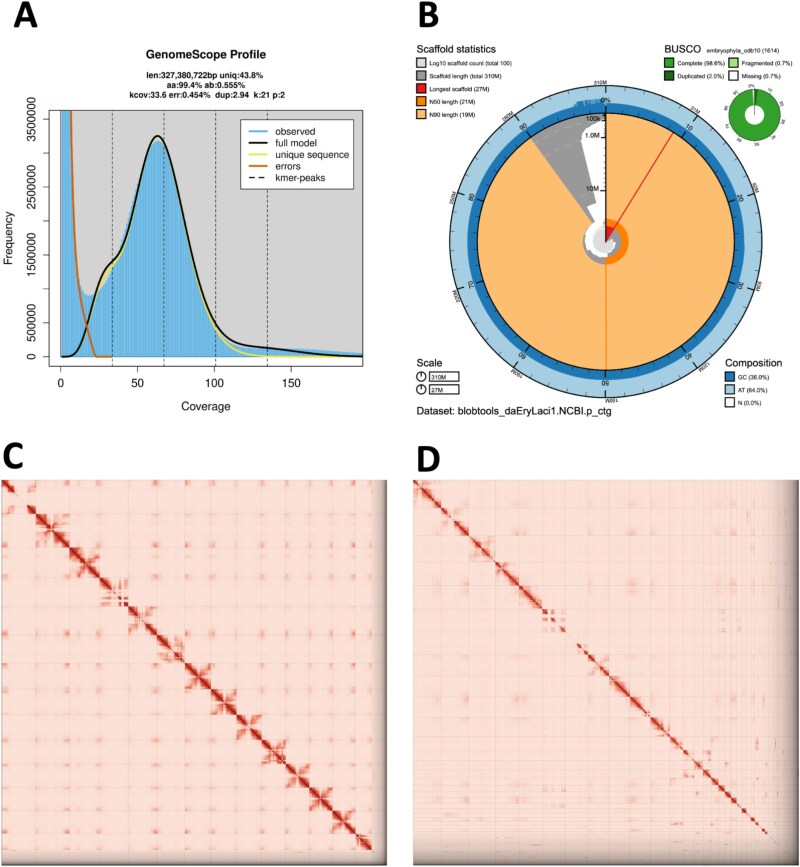
*Mimulus laciniatus* genome metrics. A) K-mer spectrum of Omni-C short read data without adapters using Genomescope 2.0. A single peak consistent with a selfer. B) BlobToolKit snail plot showing visualization of metrics of the primary assembly (daEryLaci1.0.p). C,D) Hi-C contact maps for the primary (C) and alternate (D) genome.

The Omni-C library generated 278.87 million read pairs, and the PacBio HiFi library generated 1.99 million reads. The PacBio HiFi sequences yielded ~95× genome coverage and had an N50 read length of 16,599 bp; a minimum read length of 137 bp; a mean read length of 15,173 bp; and a maximum read length of 55,779 bp (see Fig. S1 for read length distribution). Based on k-mer analysis of both short- and long-read data, we estimated a genome size of ~ 317.72 Mb. The estimations vary depending on the dataset used. Omni-C short-read data estimated a heterozygous rate of 0.56%, a sequencing error of 0.454%, and a genome size of 327.38 Mb (Fig. 2A), while the PacBio HiFi long-read data estimated a heterozygous rate of 0.26%, a sequencing error of 0.124, and a genome size of 308.07 Mb (Fig. S2a). Using default parameters for these estimations shows poor model fitting (Table S2), especially for the long-read data, so we modified the parameterization of the underlying model by using l = 50 (corresponding to the 1n peak, which is half of the coverage of our major peak; Fig. S2).

### Nuclear genome assembly

The final genome assembly (daEryLaci1) consists of two phased haplotypes. Both assemblies are similar in size, but not equal to the estimated genome size from GenomeScope2.0, as has been observed in other taxa (see e.g. Pflug et al. 2020).

The primary haplotype assembly (daEryLaci1.0.p) consists of 104 scaffolds spanning 309.97 Mb with a contig N50 of 11.09 Mb, a scaffold N50 of 20.99 Mb, the largest contig size of 24.29 Mb, and the largest scaffold size of 27.39 Mb. The alternate haplotype assembly (daEryLaci1.0.a) consists of 194 scaffolds spanning 213.85 Mb with a contig N50 of 2.36 Mb, a scaffold N50 of 11.74 Mb, the largest contig size of 9.13 Mb and the largest scaffold size of 15.56 Mb. The primary haplotype assembly has a BUSCO completeness score for the Embryophyta gene set of 98.6%, a base pair quality value (QV) of 61.08, a kmer completeness of 98.83%, and a frameshift indel QV of 51.35. The alternate haplotype assembly has a BUSCO completeness score for the Embryophyta gene set of 65.8%, a base pair QV of 60.3, a kmer completeness of 62.46%, and a frameshift indel QV of 49.71.

During manual curation we made a total of 86 joins (56 on the primary haplotype and 30 on the alternate haplotype) and 26 breaks (11 on the primary haplotype and 15 on the alternate haplotype) based on the Omni-C contact map signal. We closed a total of 18 gaps (7 on the primary haplotype and 11 on the alternate haplotype) and filtered out 5 contigs corresponding to chloroplast contamination. No other contigs were removed or modified. The Omni-C contact maps show highly contiguous assemblies, with chromosome-length scaffolds (Fig. 2B). Assembly statistics are reported in Table 2 and represented graphically in Fig. 2C. We have deposited the genome assembly on NCBI GenBank (See Table 2 and Data Availability for details).

**Table 2:** Sequencing and assembly statistics, and accession numbers.

**Bio Projects and vouchers**	CCGP NCBI BioProject			PRJNA720569			
	Genera NCBI BioProject			PRJNA765633			
	Species NCBI BioProject			PRJNA777196			
	NCBI BioSample			SAMN38285827			
	Specimen identification			CCGP_10_BB_OPN_1			
	NCBI Genome accessions			**Haplotype 1**		**Haplotype 2**	
	Assembly accession			JBCDMK000000000		JBCDML000000000	
	Genome sequences			GCA_040207155.1		GCA_040207145.1	
**Genome Sequence**	PacBio HiFi reads		Run	1 PACBIO_SMRT (Sequel IIe) run: 2 M spots, 30.2G bases, 18.5Gb			
			Accession	SRX26067539			
	Omni-C Illumina reads		Run	2 ILLUMINA (Illumina NovaSeq 6000) runs: 107.1 M spots, 32.3G bases, 7.7Gb			
			Accession	SRX26067540-1			
**Genome Assembly Quality Metrics**	Assembly identifier (Quality code^c^)			daEryLaci1(7.7.P.Q49.C98)			
	HiFi Read coverage^a^			95X			
				**Haplotype 1**		**Haplotype 2**	
	Number of contigs			148		316	
	Contig N50 (bp)			11,090,440		2,363,584	
	Contig NG50^a^			11,090,440		838,590	
	Longest Contigs			24,297,547		9,135,367	
	Number of scaffolds			104		194	
	Scaffold N50			20,993,862		11,746,579	
	Scaffold NG50^a^			20,993,862		4,460,205	
	Largest scaffold			27,399,035		15,572,277	
	Size of final assembly			309,971,392		213,857,377	
	Phased block NG50^a^			11,220,445		1,195,452	
	Gaps per Gbp (# Gaps)			142(44)		570(122)	
	Indel QV (Frame shift)			51.35164467		49.71322245	
	Base pair QV			61.0895		60.3083	
				Full assembly = 60.7531			
	k-mer completeness			98.8371		62.4651	
				Full assembly = 99.2715			
	BUSCO completeness^d^ (embryophyta) *n* = 1614		**C**	**S**	**D**	**F**	**M**
		H1^b^	98.60%	96.60%	2.00%	0.70%	0.70%
		H2^b^	65.80%	63.60%	2.20%	1.10%	33.10%
	Organelles		1 Partial chloroplast sequence			JBCDMK010000104.1	

^a^Read coverage and NGx statistics have been calculated based on the genome size of 317.72 Mb. The genome size is the average of the estimated genome sizes from PacBio HiFi reads and Omni-C Illumina short reads. ^b^(H1) Haplotype 1 and (H2) Haplotype 2 assembly values. ^c^Assembly quality code x.y.P.Q.C derived notation, from (Rhie et al. 2021). x = log10[contig NG50]; y = log10[scaffold NG50]; P = log10 [phased block NG50]; Q = Phred base accuracy QV (Quality value); C = % genome represented by the first “n” scaffolds, following a known karyotype for this species of 2n = 92 (Genome on a Tree, query (*Mimulus laciniatus*); Challis et al. 2023). Quality code for all the assembly denoted by primary assembly (daEryLaci1.0.p). ^d^BUSCO Scores. Complete BUSCOs (C). Complete and single-copy BUSCOs (S). Complete and duplicated BUSCOs (D). Fragmented BUSCOs (F). Missing BUSCOs (M).

### Chloroplast genome assembly

We assembled a partial chloroplast genome for *M. laciniatus* with Oatk. The final chloroplast sequence has a size of 155,214 bp, with base composition of A = 30.69%, C = 19.18%, G = 18.54%, T = 31.54%.

## Discussion

### Genome size distribution in the monkeyflowers

The assembly of the *M. laciniatus* genome represents the first reference genome assembly from a narrowly distributed member of the larger Phrymaceae family. The genome makes *M. laciniatus* the 11th member of genus *Mimulus* (sensu lato) and the 6th in section *Simiola* (pre Barker et al. 2012 taxonomic revision; Table 3) with a reference genome assembly. While we did not obtain a chromosome-level assembly, the first 14 scaffolds are by far the largest (Fig. 2C), consistent with prior chromosome counts (2n = 28; Vickery 1995). Of the other plant genomes published in the CCGP, spanning liverworts, ferns and vascular plants, the *M. laciniatus* genome is the smallest to date (Anghel et al. 2022, Chaturvedi et al. 2024, González-Ramírez et al. 2024, Song et al. 2025). The genome size of the primary assembly is 309.97 Mb (Fig. S2), consistent with prior flow cytometry-based estimates (i.e. 360 to 368 Mb; Ferris et al. 2014). Our genome size range falls well within the range of other genomes in section *Simiola* (average = 338 Mb; Table 3). Genome sizes, of the assemblies or as determined by flow cytometry, are relatively stable in the genus overall (Range: 207 to 630 Mb), with the exceptions of recent allotetraploid *Mimulus sookensis* (574 Mb; Whitener et al. 2024), the allotetraploid *Mimulus luteus var. luteus* (599 Mb; Cooley et al. 2022), and *Mimulus ringens* (630 Mb; Bai et al. 2012). *Mimulus luteus var. luteus* belongs to a larger clade that experienced an ancestral polyploidization event, resulting in an approximate doubling of the base chromosome number (*n* = 30; Beardsley et al. 2004). In contrast, *M. ringens,* which is not known to be a polyploid and has a smaller but variable base chromosome number (*n* = 8, 11, 12; Iyer 1980, Vickery 1995), is distantly related to the species in section *Simiola*. Nonetheless, the sequenced monkeyflower genomes likely represent a bias towards diploids, as polyploidy is common especially in section *Simiola* (Vickery 1995; Beardsley et al. 2004).

**Table 3:** Genome sizes in *Mimulus.*

**Section**	**Species**	**Population/identifier**	**Size (MB)**	**Reference**
***Simolus*** **(syn. *Simiola*)**	*Mimulus laciniatus* (syn. *Erytrhanthe lacinata*)	OPN	309.97	This study
	*Mimulus nasutus* (syn. *Erytrhanthe nasutua*)	SF	312.9	Lovell et al. 2025
	*Mimulus tilingii* (syn. *Erythranthe tillingii*)	LVR1.1	315.4	Lovell et al. 2025
	*Mimulus guttatus* (syn. *Erythranthe microphylla*)^b^	IM767 (V2)	314.6	Lovell et al. 2025
	*Mimulus guttatus* (syn. *Erythranthe microphylla*)^b^	IM62 (V3)	339.7	Lovell et al. 2025
	*Mimulus guttatus* (syn. *Erythranthe microphylla*)^b^	IM155	295	Veltsos and Kelly 2024
	*Mimulus guttatus* (syn. *Erythranthe microphylla*)^b^	IM444	323	Veltsos and Kelly 2024
	*Mimulus guttatus* (syn. *Erythranthe microphylla*)^b^	IM502	263	Veltsos and Kelly 2024
	*Mimulus guttatus* (syn. *Erythranthe microphylla*)^b^	IM541	327	Veltsos and Kelly 2024
	*Mimulus guttatus* (syn. *Erythranthe microphylla*)^b^	IM664	300	Veltsos and Kelly 2024
	*Mimulus guttatus* (syn. *Erythranthe microphylla*)^b^	IM909	311	Veltsos and Kelly 2024
	*Mimulus guttatus* (syn. *Erythranthe microphylla*)^b^	IM1034	325	Veltsos and Kelly 2024
	*Mimulus guttatus* (syn. *Erythranthe microphylla*)^b^	IM1192	284	Veltsos and Kelly 2024
	*Mimulus guttatus* (syn. *Erythranthe microphylla*)^b^	LMC-L1	277	Kollar et al. 2025
	*Mimulus guttatus* (syn. *Erythranthe grandis*)^b^	SWB-S1	278	Kollar et al. 2025
	*Mimulus sookensis* (no name under *Erythranthe*)	FAN	574	Whitener et al. 2024
	*Mimulus glabratus* (syn. *Erythrathe glabrata*)	–	391^a^	Bai et al. 2012
	*Mimulus luteus var. luteus* (syn. *Erythrathe lutea var. lutea*)	EY7	599	Cooley et al. 2022
***Erythranthe*** **(syn. *Erythranthe*)**	*Mimulus lewisii* (syn. *Erythranthe lewisi*i)	LF10 (V2)	423	http://mimubase.org/
	*Mimulus cardinalis* (syn. *Erythranthe cardinalis*)	CE10 (V2)	448	http://mimubase.org/
	*Mimulus parishii* (syn. *Erythranthe parishii*)	Mpar (v1.1)	380	http://mimubase.org/
	*Mimulus verbenaceus* (syn. *Erythranthe verbenacea*)	MvBL (v1.1)	453	http://mimubase.org/
***Diplacus***	*Mimulus aurantiacus ssp. puniceus* (syn. *Diplacus aurantiacus* ssp. *puniceus*)	UCSD	207	Stankowski et al. 2019
**–**	*Mimulus ringens*	–	630^a^	Bai et al. 2012

^a^Conversion of C-value estimates based on flow cytometry. ^b^See Nesom (2012) on taxonomic identity of these samples.

### Genomics provides insight into the process of speciation

*Mimulus laciniatus* has been recognized as a morphologically and ecologically distinct species, yet its speciation history has remained unclear. Differences in flowering time and habitat differentiation have been identified as important prezygotic reproductive isolation barriers between *M. laciniatus* and *M. guttatus* (Ferris et al. 2017, Tataru et al. 2023). Yet, early phylogenetic analyses with few nuclear markers were unable to resolve the evolutionary relationship of *M. laciniatus*, particularly whether or not it represents a selfing lineage derived from *M. guttatus* independently from the origin of *M. nasutus* (Ritland et al. 1993, Ferris et al. 2014). A recent study using whole genome resequencing data has found that *M. laciniatus* forms a well-supported clade with *M. nasutus*, and this clade is sister to the, previously named, “southern *M. guttatus* clade” that includes both annual and perennial *M. guttatus* populations (Ivey et al. 2023)*.* Additional investigation into these relationships is needed, however, to account for any complicating signals resulting from historic and ongoing introgression; natural hybrid zones between *M. laciniatus*, *M. nasutus*, *M. guttatus* likely introduce phylogenetic ambiguity (Vickery 1978; Tataru et al. 2025). The accession we have sequenced, OPN, comes from a population that is not sympatric with either *M. nasutus* or *M. gutattus*. Thus, the new *M. laciniatus* genome we have assembled provides an excellent resource that may be used in conjunction with the newly published *M. nasutus* (Lovell et al. 2025) and southern *M. guttatus* clade (Kollar et al. 2025) genome assemblies to continue disentangling the complicated evolutionary history of the yellow monkeyflower species complex as well as to investigate how gene flow and introgression have contributed to differentiation and homogenization of these lineages.

### Importance of conservation strategies tailored to endemics of the CA-FP

In an era defined by rapid biodiversity loss and climate instability, understanding how species adapt at the genomic level has become increasingly crucial for effective conservation. This new *M. laciniatus* reference genome provides a foundation for future evolutionary and conservation genetics studies to uncover genetic mechanisms underpinning resilience in extreme environments. Due to its restricted range and specialized habitat requirements, *M. laciniatus* faces inherent vulnerability to climate change, reflected by its automatic classification as California Rare Plant Rank 4. Even so, *M. laciniatus* populations possess adaptive variation that can respond to strong selection pressures. For instance, resurrection experiments using seed collected before and after the multi-year drought experienced in the Western US in the 2010s found that seed from post-drought collections germinated more quickly than seed from pre-drought collections, indicative of adaptation for drought escape (Dickman et al. 2019). Climate conditions also appear to affect the extent of gene flow with other taxa. The strength of reproductive isolation between *M. laciniatus* and *M. guttatus* is stronger in drought years compared to wetter years (Tataru et al. 2023), suggesting that drought years promote *M. laciniatus* to differentiate more from *M. guttatus*, but also limiting potential contributions of adaptive introgression to genetic or evolutionary rescue. Understanding the phenotypic and genomic diversity of *M. laciniatus* is therefore critical for predicting evolutionary and ecological responses to environmental change and guiding conservation interventions. This reference genome will allow evolutionary and landscape genomics studies that can identify adaptive genetic variants or centers of genetic diversity and guide actions such as habitat preservation, assisted migration, and genetic rescue (Shay et al. 2021, Fiedler et al. 2022; Shaffer et al. 2022). Such insights may be extendable to the > 40 additional monkeyflowers (*Mimulus* sensu lato) in the California Rare Plant Inventory (Table S1) or to California endemic plants more broadly.

## Supplementary Material

Supplementary_Figures_Revised_esaf059

Table_S1_Revised_esaf059

Table_S2_Revised_esaf059

## Data Availability

Data generated for this study are available under NCBI BioProject PRJNA777196. Raw sequencing data for sample OPN (NCBI BioSample SAMN38285827) are deposited in the NCBI Short Read Archive (SRA) under SRR30645747 for PacBio HiFi sequencing data, and SRR30645745,SRR30645746 for the Omni-C Illumina sequencing data. GenBank accessions for both primary and alternate assemblies are GCA_040207155.1 and GCA_040207145.1; and for genome sequences JBCDMK000000000 and JBCDML000000000. The GenBank genome assembly for the chloroplast genome is JBCDMK010000104.1. Assembly scripts and other data for the analyses presented can be found at the following GitHub repository: www.github.com/ccgproject/ccgp_assembly.
