# Supplementary material for: The reference genome of a Sierra Nevada endemic, the cut-leaved monkeyflower, Mimulus laciniatus (syn. Erythranthe lacinata)
Source: J Hered. 2025 Aug 28;117(2):318–28. doi: 10.1093/jhered/esaf059 (PMC13017869; doi:10.1093/jhered/esaf059)
Supplement: Supplementary_Figures_Revised_esaf059 [file supplementary_figures_revised_esaf059.docx]

**Supplementary Figure 1**
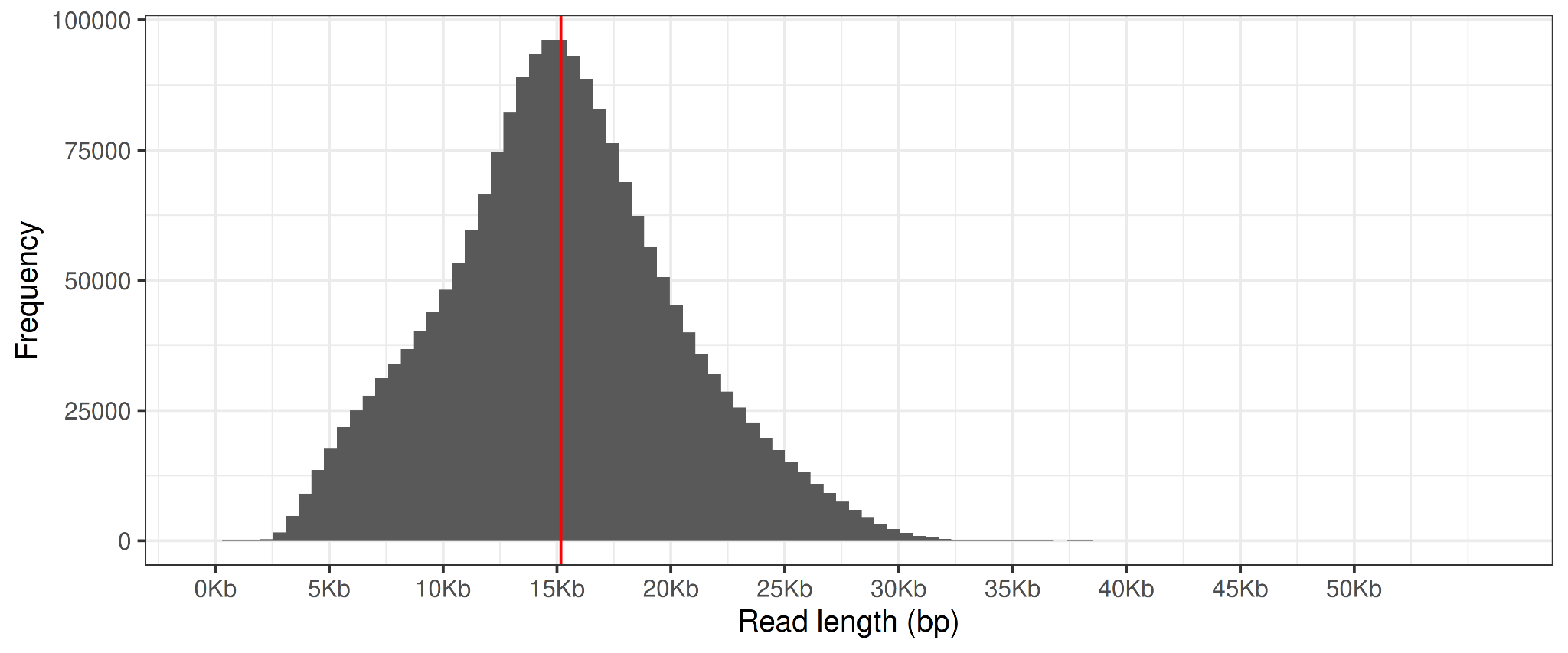


**Fig. S1** Read length distribution of the PacBio HiFi reads

Vertical red line shows average read length.

**Supplementary Figure 2**


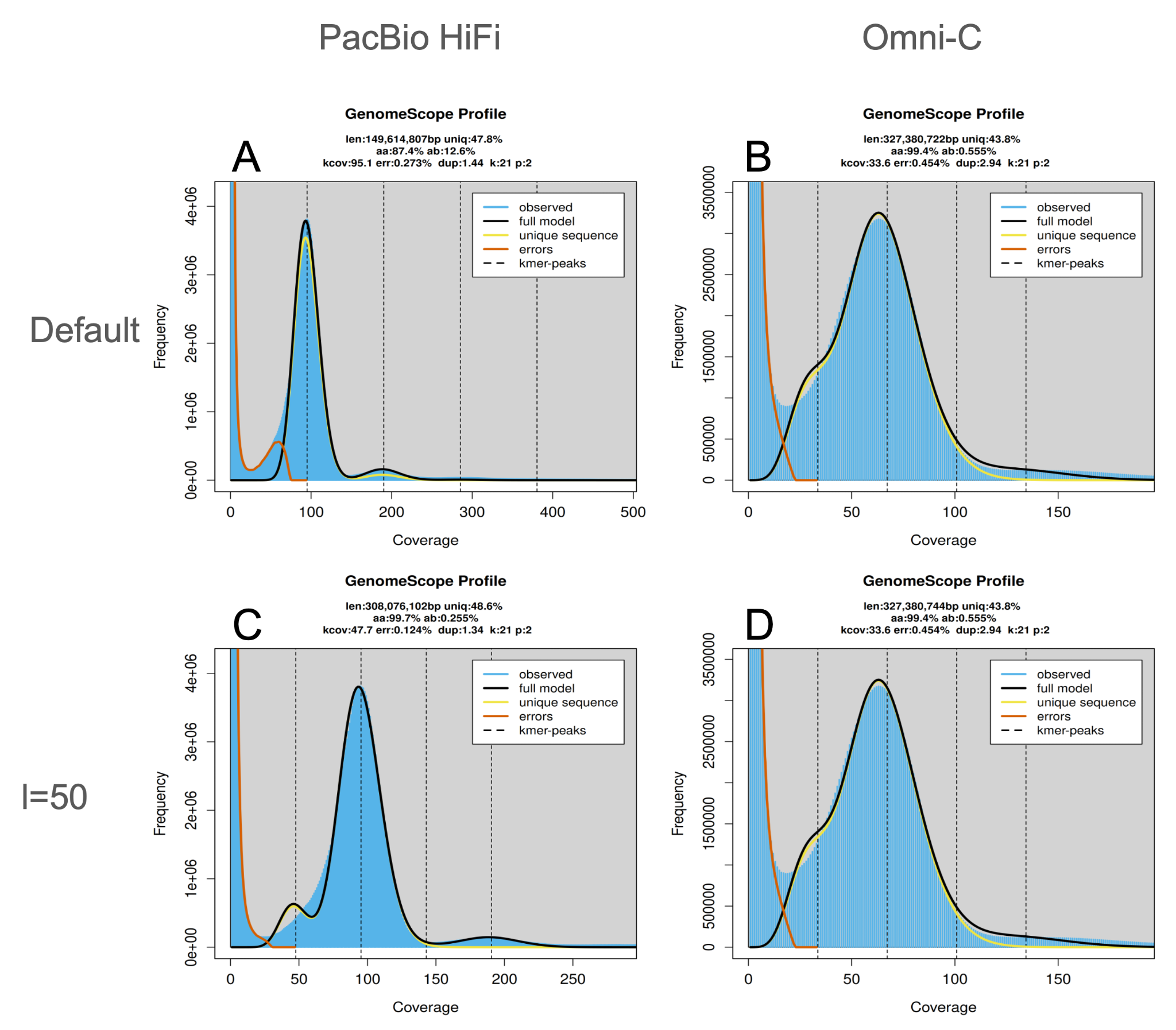


a

**Fig. S2** GenomeScope models

K-mer spectra of A,C) PacBio HiFi long-reads (without adapters) and B,D) Omni-C Illumina short-reads (without adapters) using GenomeScope 2.0 under A,B) default parameters or C,D) and under l=50 (see Results).
