# Supplementary material for: The reference genome of a Sierra Nevada endemic, the cut-leaved monkeyflower, Mimulus laciniatus (syn. Erythranthe lacinata)
Source: J Hered. 2025 Aug 28;117(2):318–28. doi: 10.1093/jhered/esaf059 (PMC13017869; doi:10.1093/jhered/esaf059)
Supplement: Table_S1_Revised_esaf059 [file table_s1_revised_esaf059.docx]

| **Table S1 - Rare California Monkeyflowers** | | | | | | | | |
| --- | --- | --- | --- | --- | --- | --- | --- | --- |
| Exported from California Native Plant Society Rare Plant Inventory database on May 2nd 2025 (https://rareplants.cnps.org). Taxa in section *Mimulus* section *Simiolus* are highlighted in gray. | | | | | | | | |
| **Species name (Pre Barker Taxonomy)** | **Species name** | **CA Endemic** | **CA Rare Plant Ranks** | **Lifeform** | **Habitat** | **Microhabitat** | DateAdded | LastUpdate |
| *Mimulus aridus, Mimulus aurantiacus var. aridus* | *Diplacus aridus* | FALSE | 4.3 | perennial evergreen shrub | Chaparral (rocky), Sonoran desert scrub |  | 1/1/1984 | 12/6/2022 |
| *Mimulus brandegeei* | *Diplacus brandegeei* | FALSE | 1A | annual herb | Coastal scrub, Valley and foothill grassland | Rocky | 1/1/1974 | 8/6/2024 |
| *Mimulus clevelandii* | *Diplacus clevelandii* | FALSE | 4.2 | perennial rhizomatous herb | Chaparral, Cismontane woodland, Lower montane coniferous forest | Disturbed areas (often), Gabbroic, Openings, Rocky | 1/1/1980 | 12/9/2021 |
| *-* | *Diplacus cusickioides* | FALSE | 2B.3 | annual herb | Great Basin scrub, Lower montane coniferous forest | Gravelly, Roadsides, Scree, Volcanic | 1/1/2001 | 12/9/2021 |
| *Mimulus johnstonii* | *Diplacus johnstonii* | TRUE | 4.3 | annual herb | Lower montane coniferous forest (disturbed areas, gravelly, roadsides, rocky, scree) |  | 1/1/2001 | 9/27/2021 |
| *Mimulus mohavensis* | *Diplacus mohavensis* | TRUE | 1B.2 | annual herb | Joshua tree "woodland", Mojavean desert scrub | Gravelly (sometimes), Sandy (sometimes), Washes (often) | 1/1/1988 | 12/9/2021 |
| *Mimulus parryi* | *Diplacus parryi* | FALSE | 2B.3 | annual herb | Great Basin scrub |  | 1/1/2001 | 5/26/2021 |
| *Mimulus flemingii, Mimulus parviflorus* | *Diplacus parviflorus* | TRUE | 4.3 | perennial evergreen shrub | Chaparral, Closed-cone coniferous forest, Coastal bluff scrub, Coastal scrub | Rocky | 1/1/1988 | 12/6/2022 |
| *Mimulus pictus* | *Diplacus pictus* | TRUE | 1B.2 | annual herb | Broadleafed upland forest, Cismontane woodland | Disturbed areas, Granitic | 1/1/1974 | 12/9/2021 |
| *Mimulus pulchellus* | *Diplacus pulchellus* | TRUE | 1B.2 | annual herb | Lower montane coniferous forest, Meadows and seeps | Clay, Disturbed areas (often), Vernally Mesic | 1/1/1974 | 12/6/2022 |
| *Mimulus pygmaeus* | *Diplacus pygmaeus* | FALSE | 4.2 | annual herb | Great Basin scrub, Lower montane coniferous forest, Meadows and seeps, Pinyon and juniper woodland | Clay, Streambanks, Vernally Mesic, Volcanic | 1/1/1974 | 12/9/2021 |
| *Mimulus rupicola* | *Diplacus rupicola* | TRUE | 4.3 | perennial herb | Mojavean desert scrub (carbonate, rocky) |  | 1/1/1974 | 11/5/2021 |
| *Mimulus traskiae* | *Diplacus traskiae* | TRUE | 1A | annual herb | Coastal scrub |  | 1/1/1974 | 5/26/2021 |
| *Mimulus fremontii var. vandenbergensis* | *Diplacus vandenbergensis* | TRUE | 1B.1 | annual herb | Chaparral, Cismontane woodland, Coastal dunes | Disturbed areas (often), Sandy | 6/7/2006 | 5/2/2023 |
| *Mimulus acutidens* | *Erythranthe acutidens* | TRUE | 3 | annual herb | Cismontane woodland, Lower montane coniferous forest |  | 1/1/1974 | 2/1/2022 |
| *-* | *Erythranthe angulosa* | TRUE | 3 | annual herb | Great Basin scrub | Granitic, Rocky | 6/21/2022 | 3/6/2024 |
| *-* | *Erythranthe bergeri* | TRUE | 3 | annual herb | Great Basin scrub | Granitic, Rocky, Vernally Mesic | 6/21/2022 | 3/6/2024 |
| *-* | *Erythranthe calcicola* | FALSE | 1B.3 | annual herb | Joshua tree "woodland", Mojavean desert scrub, Pinyon and juniper woodland | Carbonate (usually), Slopes (usually), Talus (usually) | 6/24/2013 | 12/9/2021 |
| *-* | *Erythranthe carsonensis* | FALSE | 1B.1 | annual herb | Great Basin scrub (openings) | Granitic | 6/19/2013 | 12/9/2021 |
| *Mimulus diffusus* | *Erythranthe diffusa* | FALSE | 4.3 | annual herb | Chaparral, Lower montane coniferous forest | Gravelly (sometimes), Sandy (sometimes) | 1/1/1974 | 12/9/2021 |
| *Mimulus exiguus* | *Erythranthe exigua* | FALSE | 1B.2 | annual herb | Meadows and seeps, Pebble (Pavement) plain, Upper montane coniferous forest | Clay, Mesic | 1/1/1974 | 6/4/2024 |
| *Mimulus biolettii, Mimulus filicaulis* | *Erythranthe filicaulis* | TRUE | 1B.2 | annual herb | Cismontane woodland, Lower montane coniferous forest, Meadows and seeps, Upper montane coniferous forest | Vernally Mesic | 1/1/1974 | 10/4/2022 |
| *-* | *Erythranthe filicifolia* | TRUE | 1B.2 | annual herb | Chaparral, Lower montane coniferous forest, Meadows and seeps (ephemeral) | Granitic, Seeps | 5/10/2017 | 6/8/2022 |
| *Mimulus glaucescens* | *Erythranthe glaucescens* | TRUE | 4.3 | annual herb | Chaparral, Cismontane woodland, Lower montane coniferous forest, Valley and foothill grassland | Seeps, Serpentine, Streambanks (sometimes) | 1/1/1974 | 12/9/2021 |
| *Mimulus gracilipes* | *Erythranthe gracilipes* | TRUE | 1B.2 | annual herb | Chaparral, Cismontane woodland, Lower montane coniferous forest | Burned areas (often), Decomposed granitic, Disturbed areas (often) | 1/1/1974 | 11/5/2024 |
| *Mimulus grayi* | *Erythranthe grayi* | TRUE | 4.3 | annual herb | Lower montane coniferous forest, Upper montane coniferous forest | Mesic | 1/1/1974 | 12/9/2021 |
| *-* | *Erythranthe hardhamiae* | TRUE | 1B.1 | annual herb | Chaparral (openings) | Sandstone, Sandy, Serpentine (sometimes) | 7/2/2013 | 12/9/2021 |
| *Mimulus inconspicuus* | *Erythranthe inconspicua* | TRUE | 4.3 | annual herb | Chaparral, Cismontane woodland, Lower montane coniferous forest | Mesic | 1/1/1974 | 12/9/2021 |
| *Mimulus evanescens* | *Erythranthe inflatula* | FALSE | 1B.2 | annual herb | Great Basin scrub, Lower montane coniferous forest, Pinyon and juniper woodland | Gravelly (sometimes), Rocky (sometimes), Vernally Mesic | 1/1/2001 | 12/9/2021 |
| *Mimulus laciniatus* | *Erythranthe laciniata* | TRUE | 4.3 | annual herb | Chaparral, Lower montane coniferous forest, Upper montane coniferous forest | Granitic, Mesic | 1/1/1974 | 12/9/2021 |
| *Mimulus whipplei* | *Erythranthe marmorata* | TRUE | 1B.1 | annual herb | Cismontane woodland, Lower montane coniferous forest |  | 1/1/1974 | 11/5/2024 |
| *Mimulus norrisii* | *Erythranthe norrisii* | TRUE | 1B.3 | annual herb | Chaparral, Cismontane woodland | Carbonate, Rocky | 1/1/1984 | 12/9/2021 |
| *Mimulus nudatus* | *Erythranthe nudata* | TRUE | 4.3 | annual herb | Chaparral, Cismontane woodland | Seeps, Serpentine | 1/1/1974 | 12/9/2021 |
| *-* | *Erythranthe percaulis* | TRUE | 1B.1 | annual herb | Chaparral, Lower montane coniferous forest (openings) | Roadsides, Rocky, Seeps, Serpentine, Slopes | 4/25/2017 | 12/6/2022 |
| *Mimulus purpureus* | *Erythranthe purpurea* | FALSE | 1B.2 | annual herb | Meadows and seeps, Pebble (Pavement) plain, Upper montane coniferous forest |  | 1/1/1974 | 10/4/2021 |
| *-* | *Erythranthe rhodopetra* | TRUE | 1B.1 | annual herb | Mojavean desert scrub | Sandy, Washes | 7/8/2013 | 12/9/2021 |
| *-* | *Erythranthe serpentinicola* | TRUE | 1B.1 | annual herb | Chaparral (openings), Meadows and seeps (edges) | Mesic, Openings, Rocky, Serpentine | 3/30/2022 | 9/6/2022 |
| *Mimulus shevockii* | *Erythranthe shevockii* | TRUE | 1B.1 | annual herb | Joshua tree "woodland", Pinyon and juniper woodland | Granitic (sometimes), Gravelly (sometimes), Metamorphic (sometimes), Sandy (sometimes) | 1/1/1984 | 12/9/2021 |
| *-* | *Erythranthe sierrae* | TRUE | 4.2 | annual herb | Cismontane woodland (openings), Lower montane coniferous forest (openings), Meadows and seeps (dry) | Granitic (usually), Gravelly (sometimes), Sandy (usually), Streambanks, Vernally Mesic | 10/2/2013 | 12/9/2021 |
| *-* | *Erythranthe taylorii* | TRUE | 1B.1 | annual herb | Cismontane woodland, Lower montane coniferous forest | Carbonate, Openings, Rocky | 10/16/2013 | 12/6/2022 |
| *-* | *Erythranthe trinitiensis* | TRUE | 1B.3 | annual herb | Cismontane woodland, Lower montane coniferous forest, Meadows and seeps, Upper montane coniferous forest | Roadsides (often), Serpentine (often) | 1/3/2014 | 12/6/2022 |
| *Mimulus glabratus ssp. utahensis* | *Erythranthe utahensis* | FALSE | 2B.1 | perennial rhizomatous herb | Meadows and seeps, Pinyon and juniper woodland |  | 1/1/1980 | 5/26/2021 |
| *-* | *Mimulus rattanii ssp. decurtatus* | TRUE | 4.2 | annual herb | Chaparral, Lower montane coniferous forest | Gravelly, Lake Margins | 1/1/1974 | 4/2/2024 |
| *-* | *Mimulus subsecundus* | TRUE | 4.3 | annual herb | Chaparral, Lower montane coniferous forest |  | 1/1/1974 | 4/2/2024 |
