# Supplementary material for: The reference genome of a Sierra Nevada endemic, the cut-leaved monkeyflower, Mimulus laciniatus (syn. Erythranthe lacinata)
Source: J Hered. 2025 Aug 28;117(2):318–28. doi: 10.1093/jhered/esaf059 (PMC13017869; doi:10.1093/jhered/esaf059)
Supplement: Table_S2_Revised_esaf059 [file table_s2_revised_esaf059.docx]

| **Table S2 - GenomeScope model parameters comparison** | | | | | | | | | | | | | |
| --- | --- | --- | --- | --- | --- | --- | --- | --- | --- | --- | --- | --- | --- |
| **Sequencing data** | **ploidy** | **k** | **l** | **aa** | **ab** | **Heterozygosity rate** | **kcov** | **Error rate** | **Model fit (score)** | **Model fit (min)** | **Model fit (max)** | **Estimated genome length (bp)** |  |
| PacBio HiFi Long reads | 2 | 21 | default | 87.40% | 12.60% | 0.126 | 95 | 0.273% | 1.44 | 48.65% | 84.70% | 149,614,807 |  |
| PacBio HiFi Long reads | 2 | 21 | 50 | 99.70% | 0.26% | 0.00255 | 48 | 0.124% | 1.34 | 49.99% | 91.99% | 308,076,102 |  |
| Omni-C Illumina short-reads | 2 | 21 | default | 99.40% | 0.56% | 0.00555 | 34 | 0.454% | 2.94 | 45.82% | 94.48% | 327,380,722 |  |
| Omni-C Illumina short-reads | 2 | 21 | 50 | 99.40% | 0.56% | 0.00555 | 34 | 0.454% | 2.94 | 45.82% | 94.48% | 327,380,744 |  |
